# Seizures in adults with suspected central nervous system infection

**DOI:** 10.1186/s12883-022-02927-4

**Published:** 2022-11-14

**Authors:** Sabine E. Olie, Ingeborg E. van Zeggeren, Liora ter Horst, I.-PACE Study Group, J. Citroen, B. M. van Geel, S. G. B. Heckenberg, K. Jellema, M. I. Kester, J. Killestein, B. B. Mook, M. J. Titulaer, K. E. B. van Veen, C. V. M. Verschuur, Diederik van de Beek, Matthijs C. Brouwer

**Affiliations:** 1 Department of Neurology, Amsterdam UMC, University of Amsterdam, Amsterdam Neuroscience, Meibergdreef 9, 1105 AZ Amsterdam, the Netherlands; 2 Department of Neurology, Amsterdam UMC, Vrije Universiteit Amsterdam, Amsterdam Neuroscience, De Boelelaan 1117, Amsterdam, the Netherlands

**Keywords:** CNS infection, Cerebrospinal fluid, Epilepsy, Diagnostic accuracy

## Abstract

**Background:**

Seizures can be part of the clinical presentation of central nervous system (CNS) infections. We describe patients suspected of a neurological infection who present with a seizure and study diagnostic accuracy of clinical and laboratory features predictive of CNS infection in this population.

**Methods:**

We analyzed all consecutive patients presenting with a seizure from two prospective Dutch cohort studies, in which patients were included who underwent cerebrospinal fluid (CSF) examination because of the suspicion of a CNS infection.

**Results:**

Of 900 episodes of suspected CNS infection, 124 (14%) presented with a seizure. The median age in these 124 episodes was 60 years (IQR 45–71) and 53% of patients was female. CSF examination showed a leukocyte count ≥ 5/mm^3^ in 41% of episodes. A CNS infection was diagnosed in 27 of 124 episodes (22%), a CNS inflammatory disorder in 8 (6%) episodes, a systemic infection in 10 (8%), other neurological disease in 77 (62%) and in 2 (2%) episodes another systemic disease was diagnosed. Diagnostic accuracy of clinical and laboratory characteristics for the diagnosis of CNS infection in this population was low. CSF leukocyte count was the best predictor for CNS infection in patients with suspected CNS infection presenting with a seizure (area under the curve 0.94, [95% CI 0.88 – 1.00]).

**Conclusions:**

Clinical and laboratory features fail to distinguish CNS infections from other causes of seizures in patients with a suspected CNS infection. CSF leukocyte count is the best predictor for the diagnosis of CNS infection in this population.

## Background

Patients suspected of a central nervous system (CNS) infection often pose a diagnostic dilemma [1]. The differential diagnosis can be broad, and the diagnostic accuracy of clinical and laboratory features in this group is insufficient to differentiate between neurological infections and other diagnoses [2]. Seizures can be part of the clinical presentation of a CNS infection and have been described in approximately a quarter of all patients [3], with frequencies ranging from 7 to 28% in bacterial meningitis [4–8] and from 40 to 75% in herpes simplex virus (HSV) encephalitis.[9–12] Pediatric studies have focused on how to identify patients with a CNS infection from cohorts of patients presenting with a first seizure and fever [13, 14]. A meta-analysis of 1996 patients showed that the risk of bacterial meningitis in this population is low (2.6%) [15]. However, characteristics predictive for bacterial meningitis could not be identified. Studies also show that an elevated CSF leukocyte count, previously identified as the strongest predictor of CNS infections, can be found in 10% of children presenting with a seizure and no CNS infection [16]. In this study we aim to identify the diagnostic accuracy of clinical and laboratory characteristics for the diagnosis of CNS infection in patients suspected of a CNS infection who present with a seizure.

## Methods

### Patient inclusion and data collection

We included adult patients (≥ 16 years of age) with a clinically suspected CNS infection who underwent CSF examination. Patients were included in two prospective cohort studies. The first study (September 2012 – February 2015) was a single center pilot study. The second study is an ongoing (September 2017 – now) multicenter cohort study in the Netherlands. Patients who were eligible for inclusion were reported to the investigators by the treating physician or identified during morning rounds. We obtained written informed consent from all participating patients or their legal representatives. We excluded patients with recent (≤ 1 month) head injury or neurosurgery, and patients with neurosurgical devices. Online case record forms (CRF) were used to collect data on patients’ characteristics and medical history, symptoms at presentation, laboratory results, radiological imaging, antibiotic or antiviral treatment, and outcome. The CRF included a standard question on the presence or absence of seizures on admission, as well as the type of seizure.

All patient data was rendered anonymous and the study was carried out in accordance with Dutch privacy legislation. The study was approved by the biobank ethics committee of the Amsterdam UMC, location AMC, Amsterdam, The Netherlands (number BTC AMC2014_290).

### Procedures and definitions

Seizures were classified according to seizure type into focal onset, generalized onset or unknown onset using the International League Against Epilepsy classification [17]. Seizures without an identifiable cause were defined as seizures of uncertain etiology, in literature also known as idiopathic seizures, cryptogenic seizures or unprovoked seizures. Hospital-acquired disease was defined as an episode of (suspected) CNS infection occurring during admission (> 48 h after presentation) or within one week after discharge. Other episodes were considered community-acquired. Patients were considered to be immunocompromised if they were using immunosuppressive drugs or had a medical history of diabetes mellitus, auto-immune disease, alcoholism, human immunodeficiency virus (HIV) infection or splenectomy. The Glasgow Coma Scale (GCS) score was used to assess level of consciousness at presentation [18]. Patients with a GCS score of ≤ 14 were considered to have an altered mental status, and a GCS score of ≤ 8 indicated coma. In patients who underwent cranial imaging, modality (CT or MRI) and cranial abnormalities were documented in the CRF. Glasgow Outcome Scale (GOS) was used to score the outcome at time of discharge, with scores ranging from 1 to 5, indicating the following outcome: 1 death; 2 persistent vegetative state; 3 severe disability; 4 moderate disability and 5 good recovery. A score from 1–4 on the GOS was defined as an unfavorable outcome and a score of 5 was defined as a favorable outcome [19].

### Diagnostic categorization

The final diagnosis of all episodes was classified according to the following five categories, 1) CNS infection, 2) CNS inflammation, 3) systemic infection, 4) other neurological disease, 5) other systemic disease. The rationale and methods of this categorization have been described previously [2]. Two clinicians independently categorized all episodes and differences were resolved by consultation of a third clinician. Inter-rater agreement was assessed by calculation of the kappa coefficient with a Kappa of 0.76 in the first study and 0.64 in the second study.

### Statistical analysis

Statistical analyses were conducted with the use of SPSS statistical software, version 26 (SPSS, Inc.). We used descriptive statistics for baseline characteristics with medians and interquartile range (IQR). Continuous data were compared with the used Mann–Whitney U test. For categorical data the Fisher’s exact test was used. The area under the curve (AUC) of receiver operator characteristics (ROC), sensitivity, specificity, positive predictive value (PPV) and negative predictive value (NPV) were used to evaluate diagnostic accuracy of clinical and laboratory characteristics. All tests were 2-tailed, and *P* < 0.05 was considered significant.

## Results

We included a total of 900 episodes with suspected CNS infection. Of these episodes, 124 (14%) presented with a seizure of whom 93 of 121 (77%) were evaluated at the emergency department, 12 (10%) in the intensive care unit and 16 episodes (13%) in a hospital ward. Community acquired CNS infection was suspected in 112 of 124 episodes (90%), and a nosocomial CNS infection in 12 out of 124 (10%). The median age was 60 years (IQR [45–71]) and 66 (50%) of the patients were female (Table 1). Of all episodes, 53 (43%) were immunocompromised, most often due to diabetes mellitus (23 episodes, 19%) and due to the use of immunosuppressive medication (17 episodes, 14%). A history of epilepsy was present in 31 episodes (25%), of which 11 (35%) were previously diagnosed with epileptic seizures of uncertain etiology.

**Table 1:** Clinical characteristics, laboratory parameters and outcome of 124 patients with suspected neurological infections presenting with seizures^a^

Characteristic	n/N (%)	Characteristic	n/N (%)	Characteristic	n/N (%)
Median age (IQR), years	60 (45–71)	Score Glasgow Coma Scale^b^	11 (7–14)	Outcome	
Immunocompromised state	53/124 (43)	Altered mental status ≤ 14	107/123 (86)	Death	17/124 (14)
Diabetes	23/124 (19)	Coma ≤ 8	42/123 (34)	Unfavorable	63/124 (51)
Alcoholism	14/124 (11)	Neck stiffness	11/96 (11)	Good recovery	61/124 (49)
Immunosuppressive therapy	17/124 (14)	Type of seizure		Final diagnostic category	
HIV positive	7/124 (6)	Generalized	70/104 (67)	CNS infection	27/124 (22)
History of epilepsy	31/124 (25)	Focal	20/104 (19)	CNS inflammatory disease	8/124 (6)
Symptomatic epilepsy	20/31 (65)	Both	14/104 (13)	Systemic infection	10/124 (8)
Duration of symptoms		Blood chemistry^c^		Other neurological disease	77/124 (62)
< 24 h	83/115 (72)	C-reactive protein (CRP)	10 (3 – 49)	Other systemic disease	2/124 (2)
		> 5 mg/L	72/113 (64)		
Presenting symptoms		> 40 mg/L	32/113 (26)		
Headache	33/91 (36)	Leukocytes	11.6 (7.7 – 15.2)		
Vomiting or nausea	26/94 (28)	> 10.5 × 10^9^/L	71/123 (57)		
Diarrhea	4/78 (5)	CSF examination^d^			
Clinical signs		Opening pressure (cm H_2_O)	19 (15–26)		
Fever (> 38.0)	41/122 (33)	CSF leukocytes (/mm3)	3 (3–11)		
Hypotension (diastolic BP < 50 mm Hg)	10/122 (8)	CSF leukocytes ≥ 5/mm3	51/123 (41)		
Tachycardia (HF > 120 bpm)	17/122 (14)	CSF leukocytes > 100/mm3	16/123 (13)		
		CSF protein (g/L)	0.43 (0.33–0.78)		
		CSF to blood glucose ratio	0.55 (0.44–0.65)		

^a^Data are n/N (%) or median (interquartile range)

^b^Glasgow Coma scale score was known for 123 patients

^c^CRP was known for 114 episodes, blood leukocytes for 123 episodes

^d^Lumbar puncture opening pressure was known for 80 episodes, CSF leukocyte count for 123 episodes, CSF protein concentration for 122 episodes, CSF to blood glucose ratio for 117 episodes

Symptoms were present for less than 24 h in 83 out of 115 episodes (72%). The most common presenting feature was an altered mental status (107 of 123 [86%]). Headache was reported in 33 of 124 (27%) episodes, fever in 41 of 122 (33%) and neck stiffness in 11 of 124 (9%). Focal neurological deficits were present in 53 of 124 (43%) episodes and included aphasia (15 episodes, 12%), cranial nerve palsy (11 episodes, 8%), paresis (46 episodes, 37%), ataxia (1 episode, 1%) and pathological reflexes (20 episodes, 16%).

### Ancillary investigations

Cranial imaging (Computed Tomography [CT] or Magnetic resonance imaging [MRI]) at presentation was performed in 118 out of 124 (95%) episodes and showed abnormalities in 69 of 117 (59%) scans. Non recent vascular lesions were the most common abnormality and were found in 25 of 69 (36%) scans. Other abnormalities included (semi) recent infarction (6 episodes, 9%), mastoid and sinus opacification (5 episodes, 7%), generalized edema (3 episodes, 4%) and hydrocephalus (2 episodes, 3%). Electroencephalogram (EEG) was performed during or after admission in 54 of 124 (44%) episodes and showed abnormalities consistent with epilepsy in 24 (44%) episodes.

Lumbar puncture was performed in all patients. The opening pressure was measured in 80 of 124 episodes (65%) and showed a median pressure of 19 cm H_2_O (IQR 15–26). An opening pressure of  ≥ 20 cm H_2_O was observed in 39 (49%) episodes, and in 3 (4%) episodes a pressure of ≥ 40 cm H_2_O was measured. Median CSF leukocyte count was 3/mm^3^ (IQR 3–11). Elevated leukocyte count (≥ 5mm^3^) was present in 51 of 123 (41%) episodes and 16 of 123 (13%) episodes showed a leukocyte count of > 100/mm^3^. CSF protein levels of > 0.6 g/L were present in 43 of 122 (35%) episodes and a decreased CSF to blood glucose ratio (< 0.6) was found in 72 of 117 (62%) episodes. Of all patients, 23 had a final diagnosis of epileptic seizures of uncertain etiology (19%) of which 2 (9%) had a leukocyte count ≥ 5/mm^3^, presenting with a CSF leukocyte count of 6 and 16/mm^3^ (Table 2). These elevated counts could be explained by an elevated blood leukocyte count of 28.9 × 10^9^/L, and blood admixture during the lumbar puncture resulting in an red blood cell count of 17,000/mm^3^, respectively.

**Table 2:** CSF examination in 23 patients with epileptic seizures of uncertain etiology^a^

Characteristics	n/N(%)
Opening pressure (cm H_2_O)^b^	16 (11–19)
CSF leukocytes (per mm^3^)	1 (1–2)
CSF leukocytes ≥ 5/mm^3^	2/23 (9)
CSF leukocytes > 100/mm^3^	0/23 (0)
CSF protein (g/L)	0.37 (0.33–0.42)
CSF protein > 0.6 g/L	4/23 (17)
Blood to CSF glucose ratio	0.55 (0.50–0.60)
Ratio < 0.6	16/23 (70)

^a^Data are n/N (%) or median (interquartile range)

^b^Lumbar puncture opening pressure was known for 14 episodes, CSF leukocyte count, CSF protein and CSF to blood glucose ratio for 23 episodes

CSF culture was performed in 92 of 124 (74%) episodes and was positive in 5 episodes (5%). Polymerase chain reaction (PCR) for viral and bacterial DNA in CSF was performed in 92 of 124 (74%) and was positive in 12 episodes (13%) of which 2 positive Epstein-Barr virus PCRs were judged to be clinically not relevant. CSF cultures and PCR were not performed if the suspicion of a CNS infection was no longer present after the lumbar puncture, i.e. if an alternate condition was diagnosed or if the suspicion was low prior to the lumbar puncture and the CSF examination showed no leukocytosis.

Antiviral or antibiotic treatment was started in 97 out of 124 episodes (78%). Of these, 59 received (61%) antibiotics according to bacterial meningitis protocol and 16 (16%) patients received monotherapy of acyclovir. In 25 episodes, (20%), the patients received both acyclovir and antibiotics. For 92 of 122 (75%) episodes, patients were treated with anti-epileptic drugs during admission, of which 30 out of 92 (33%) had been using antiepileptic drugs before admission.

### Final diagnosis and outcome

**Fig. 1:**
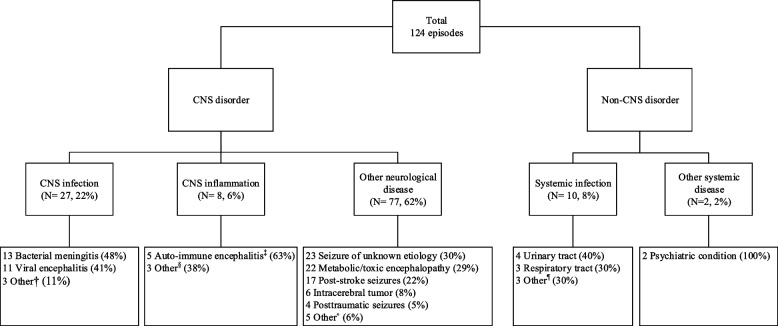
Diagnosis in patients with suspected neurological infections presenting with seizures. ^†^ 1 Neurocysticercosis, 1 cerebral toxoplasmosis, 1 cerebral Whipple’s disease, ^‡^ 1 anti-N-methyl-D-aspartate (NMDA), 1 anti-Leucine-Rich Glioma-Inactivated1 (LGI1) and 3 seronegative, ^§^ 1 cerebral vasculitis, 1 neuro systemic lupus erythematosus (SLE), 1 Acute disseminated encephalomyelitis (ADEM), ^•^ 3 status post CNS infection, 1 hypertensive encephalopathy, 1 encephalopathy due to thrombotic thrombocytopenic purpura. ^¶̊^ 1 skin/soft tissue infection, 1 abdominal infection, 1 bacteremia

A CNS infection was diagnosed in 27 of 124 episodes (22%; Table 1), most commonly bacterial meningitis (13 episodes, 48%) and viral encephalitis (11 episodes, 41%; Fig. 1). Overall, the causative pathogen was found in 17 out of 27 episodes (63%) of CNS infections. In bacterial meningitis the causative bacteria were identified in CSF (culture or PCR) or blood in 6 of 13 episodes (46%): *Streptococcus pneumoniae* in 5 episodes (38%) and *Streptococcus anginosus* in 1 episode (8%). The causative virus in viral encephalitis was found in 7 out of 11 episodes (64%), HSV in 4 episodes (50%), varicella zoster (VZV) in 2 episodes (25%) and John Cunningham (JC) virus in 1 episode (13%).

CNS inflammation was diagnosed in 8 episodes (6%), of which 5 (63%) were diagnosed with auto-immune encephalitis. Other neurological diagnosis were made in 77 (62%) episodes, most commonly seizures of uncertain etiology (23 episodes, 30%), metabolic or toxic encephalopathy (22 episodes, 29%), post-stroke seizures (17 episodes, 22%), and intracerebral tumors (6 episodes, 8%). In 10 episodes (8%) a systemic infection was diagnosed, most often urinary tract infections (4 episodes, 40%) and respiratory tract infections (3 episodes, 30%). Two episodes (2%) were diagnosed with another systemic disease. These patients initially presented with an episode highly suspicious for an epileptic seizure, but were both ultimately diagnosed with pseudo-epilepsy.

Outcome was known for all episodes: 63 patients (51%) had an unfavorable outcome, of which 17 (14%) died during admission. (Table 1, 3) An unfavorable outcome most commonly occurred in patients with CNS inflammation (8 of 8 episodes, 100%). Outcome in patients diagnosed with CNS infection did not differ from patients with another final diagnosis (*p* = 0.39).

**Table 3:** Clinical presentation, laboratory characteristics and outcome per disease category^a^

Characteristic	CNS infection (*N* = 27)	CNS inflammation (*N* = 8)	Systemic infection (*N* = 10)	Other neurological disease (*N* = 77)	Other systemic disease (*N* = 2)
Headache	10/20 (50)	5/6 (83)	2/6 (33)	15/57 (26)	1/2 (50)
Neck stiffness	5/20 (25)	3/7 (43)	2/9 (22)	1/58 (2)	0
Fever	15/27 (56)	2/7 (29)	5/10 (50)	19/76 (25)	0
Predisposing infection	5 (19)	0	2/10 (20)	4/77 (52)	0
Altered mental status	24/27 (89)	5/8 (63)	7/10 (70)	69/76 (91)	2/2 (100)
Coma	12/27 (44)	1/8 (13)	1/10 (10)	28/76 (37)	0
Blood leukocytosis (> 10.5)	15/27 (56)	4/8 (50)	7/10 (70)	44/76 (58)	1/2 (50)
CRP, median	54 (4–270)	3 (1–27)	41 (9–192)	7 (2–33)	4 (-)
CRP > 5	19/26 (73)	3/7 (43)	7/9 (78)	43/70 (61)	0
CRP > 40	13/26 (50)	1/7 (14)	5/9 (56)	13/70 (19)	0
CSF leukocyte count, median	112 (36–684)	7 (2–37)	1 (1–2)	2 (1–5)	2 (-)
≥ 5/mm3	24/26 (92)	4/8 (50)	0	23/77 (30)	0
> 100/mm3	15/26 (60)	1/8 (13)	0	0	0
Unfavorable outcome	16/27 (59)	8/8 (100)	5/10 (50)	33/77 (43)	1/2 (50)
Death	8/27 (30)	1/8 (13)	2/10 (20)	6/77 (8)	0

^a^Data are n/N (%) or median (interquartile range)

Patients presenting with a new-onset seizure were more often diagnosed with CNS infection or inflammation (31 of 93 [33%]) than patients with a history of seizures (4 of 31 episodes [13%], *p* = 0.04) (Table 4).

**Table 4:** Clinical and laboratory features, diagnostic category and outcome in 93 patients with a first seizure and 31 patients with a history of seizures^a^

Characteristics	First seizure (*N* = 93)	History of seizures (*N* = 31)	*P*-value
Age	59 (57–61)	63 (60–66)	0.954
Immunocompromised state	41/93 (44)	12/31 (39)	0.678
Duration of symptoms < 24 h	63/93 (68)	20/31 (65)	0.443
Focal neurologic deficits	32/93 (34)	21/31 (68)	0.002**
CSF leukocytes ≥ 5/mm3	28/92 (30)	13/31 (42)	1.000
Final diagnosis of CNS infection or inflammation	31/93 (33)	4/31 (13)	0.037*
Unfavorable outcome	43/93 (46)	20/31 (65)	0.098

^a^Data are n/N (%) or median (interquartile range), *P ≤ 0.05, **P ≤ 0.01

### Prediction of diagnosis – diagnostic accuracy

There were no distinctive differences between diagnostic groups with regard to clinical, laboratory and radiological features (Table 3, 5). Of all CNS infection episodes, 10 out of 20 (50%) presented with headache. Neck stiffness was found in 5 of 20 (25%) episodes of CNS infection, but was also found in CNS inflammation, systemic infections and other neurological diseases. In 15 of 27 (56%) episodes of CNS infection there was a fever upon presentation.

**Table 5:** Test characteristics of clinical and laboratory characteristics

	**Neurological infection**		**Other diagnoses**		**Sens (95%CI)**	**Spec (95%CI)**	**PPV (95%CI)**	**NPV (95%CI)**
	Present	Absent	Present	Absent				
Headache	10	10	23	48	50% (27%—73%)	68% (55% – 78%)	30% (20%—43%)	83 (75%—88%)
Nausea/vomiting	6	15	20	53	29% (11%—52%)	73% (61%—82%)	23% (12%—40%)	78% (72%—83%)
Immunocompromised	10	17	43	54	37 (19% – 57%)	56 (45%—66%)	19% (12%—29%)	76% (69%—82%)
Altered mental status (GCS ≤ 14)	24	3	83	13	89% (71%—98%)	14% (7%—22%)	22% (20%—25%)	81% (57%—94%)
Coma (GCS ≤ 8)	12	15	30	66	44% (25%—65%)	69% (58%—78%)	29% (19%—40%)	81% (75%—86%)
Neck stiffness	5	15	6	70	25% (8%—49%)	92% (84%—97%)	45% (22%—71%)	82% (78%—85%)
Generalized seizure	16	8	54	35	67% (45%-84%)	39% (29%—59%)	23% (18%—29%)	81% (70%—89%)
Diast BP < 50 mmHg	3	24	7	88	11% (2%—29%)	93% (85%—97%)	30% (11%—61%)	79% (66%—82%)
Tachycardia	7	20	10	85	26% (11%—46%)	89% (81%—95%)	41% (23%—62%)	81% (77%—84%)
Fever > 38.0 °C	15	12	26	69	56% (35%—75%)	73% (63%—81%)	37% (27%—48%)	85% (79%—89%)
Focal neurological abnormalities	8	19	45	52	30% (14%—50%)	54% (43%—64%)	15% (9%—25%)	73% (67%—79%)
Blood leukocytose (≥ 10.5)	15	12	56	40	56% (36%—75%)	42% (32%—52%)	21% (16%—28%)	77% (67%—84%)
CRP > 5 mg/L	19	7	53	34	73% (52%—88%)	39% (29%—50%)	26% (21%—32%)	83% (71%—91%)
CRP > 40 mg/L	13	13	19	68	50% (30%—70%)	78% (68%—86%)	41% (28%—54%)	84% (78%—89%)
CSF leukocytes ≥ 5/mm^3^	24	2	27	70	92% (75%—99%)	72% (62%—81%)	47% (39%—56%)	97% (90%—99%)
CSF leukocytes > 100/mm^3^	15	11	1	96	58% (37%—77%)	99% (94%—100%)	94% (67%—99%)	90% (85%—93%)
CSF protein > 0.6 g/L	18	9	25	72	67% (46%—83%)	74% (74%—83%)	42% (32%—53%)	89% (82%—93%)
CSF protein > 2 g/L	8	19	3	94	30% (14%—50%)	97% (91%—99%)	72% (43%—90%)	83% (79%—86%)
CSF pressure > 22 mm H_2_O	7	3	21	49	70% (35%—93%)	70% (58%—80%)	25% (16%—36%)	94% (86%—98%)
CSF:blood glucose ratio < 0.6	21	4	53	37	84% (64%—95%)	41% ( 31%—52%)	28% (24%—34%)	90% (78%—95%)

CSF leukocytosis ≥ 5/mm^3^ was present in 24 of 26 (92%) episodes of CNS infection, 4 of 8 (50%) in CNS inflammation and in 23 of 77 (30%) of other neurological disease episodes. CSF leukocytosis ≥ 5/mm^3^ was not present in patients with a systemic infection or other systemic disease. The specificity of CSF leukocytosis ≥ 5/mm^3^ for distinguishing all CNS disorders (CNS infection, CNS inflammation and other neurological diseases) from all non-CNS disorders (systemic infection and other systemic disease) was high, but with low sensitivity (sensitivity 46%, 95% CI 36–56%; specificity 100%, 95% CI 74–100%). CSF leukocytosis > 100/mm^3^ had a high specificity but low sensitivity for differentiating CNS infections from other diagnosis (sensitivity 58%, 95% CI 37—77%; specificity 99%, 95% CI 94–100%; Table 5). CSF leukocytosis > 100/mm^3^ was present in 15 of 26 (60%) episodes of CNS infection, and in 1 of 8 (13%) episodes in de CNS inflammation group. CSF leukocytosis > 100 mm^3^ was not present in any of the other diagnostic groups. Both patients who were diagnosed with a CNS infection but had a CSF leukocyte below the threshold of 5/mm^3^ were HIV positive, and suffered from cerebral toxoplasmosis and progressive multifocal leukoencephalopathy (CD4 count respectively 120 and 34 × 10^6/l, viral load respectively 1984 and 17,600 copies/ml).

For single predictors, the AUC for predicting CNS infection was 0.94 (95% CI 0.88 – 1.00) for CSF leukocytes, 0.81 (95% CI 0.70 – 0.91) for CSF total protein and 0.74 (95% CI 0.63 – 0.85) for CSF:blood glucose ratio. Combining these individual predictors did not substantially increase the diagnostic accuracy compared to CSF leukocyte count (AUC 0.96 [95% CI 0.93 – 1.00]).

## Discussion

Our study showed that 22% of episodes with suspected CNS infections presenting with a seizure was diagnosed with a CNS infection. The incidence of CNS infection as cause of acute symptomatic seizures has not been well established and has only been studied in retrospective cohorts studying acute symptomatic seizures. In these cohorts the proportion of patients in whom CNS infection was the cause of the seizure ranged from 15 to 28%, with a higher incidence in countries where neurotuberculosis and neurocysticercosis are endemic [20, 21]. Other common causes of acute symptomatic seizures are alcohol/drugs use or abstinence, brain tumors, neuroinflammatory diseases, traumatic head injury and cerebrovascular disease [20–23]. Differentiating between these causes can pose a diagnostic challenge. Our study shows that the diagnostic accuracy of most clinical characteristics and laboratory features for the diagnosis of CNS infection was low.

CSF leukocyte count was the best predictor for CNS infections with an AUC of 0.94, but lacked specificity. CSF leukocytosis was present in 92% of episodes with a CNS infection, but in 28% of other diagnosis as well. Only 2 patients without an elevated CSF leukocyte count were finally diagnosed with a CNS infection. Both patients were HIV infected and suffered from HIV-associated opportunistic infections. The patients in our study were diagnosed with a cerebral *Toxoplasma gondii* infection and progressive multifocal leukoencephalopathy (PML). As these infections are primarily located intracerebrally, CSF examination is often not diagnostic for these diseases as CSF parameters can be within normal limits [24–26]. A normal CSF leukocyte count in non-HIV patients with a CNS infection was not encountered, and ruled out CNS infection in our study population.

One third of patients presenting with seizures but without CNS infection had an elevated CSF leukocyte count. These patients were diagnosed with a range of different disorders, such as post-stroke epilepsy, seizures due to intracerebral tumors or metabolic disturbances. CSF leukocytosis has been reported in these conditions, independently of the presence of epileptic seizures [27–29]. In the current study, CSF changes in epileptic seizures of uncertain etiology were uncommon. Only 2 of 23 episodes with a final diagnosis of epileptic seizures of uncertain etiology had an elevated CSF leukocyte count, both of which could be explained by external factors (blood leukocytosis and blood admixture). The hypothesis that epileptic seizures of uncertain etiology cause CSF leukocytosis due to ictal activity alone has been mostly supported by studies conducted in the 1980s [30–33]. These studies found an incidence ranging from 11%-30% of CSF leukocytosis in epileptic seizures of uncertain etiology. More recent studies have shown that CSF leukocytosis in this group is very rare and that in most cases an underlying cause for the elevated leukocyte count is found [34–38]. This difference can be explained by a number of factors. First, diagnostic options when the initial studies were conducted were limited compared to today. MRI and PCR were not or only scarcely available, which might have led to an incorrect diagnosis of epileptic seizure of uncertain etiology. Furthermore, the definition of leukocytosis differed. Some studies regarded a CSF polymorphonuclear leukocyte count of > 0 as leukocytosis, [30, 31, 33] while in current practice a leukocyte count of ≥ 5 is generally defined as leukocytosis, regardless of leukocyte type [39]. This has led to an overestimation of the proportion of patients with seizure of uncertain etiology and CSF leukocytosis. Finally, inclusion and exclusion criteria were not always clear and some of the less recent studies excluded patients with symptomatic seizures, caused by infection, stroke or trauma.[31, 32] Our results confirm the more recent studies, and therefore CSF leukocytosis in patients with seizures and suspected CNS infection should prompt further search for the underlying cause as it cannot be attributed to seizure activity alone.

There were several limitations to our study. First, in our study we only included patients who underwent CSF examination. Patients presenting with a seizure in whom cranial imaging revealed a probable cause of the seizure are unlikely to undergo a lumbar puncture and were therefore not included in our study. Also, in patients presenting with a seizure without other signs of a CNS infection a lumbar puncture is not routinely performed. This means that CNS infections could have been missed. Furthermore, the presence of an epileptic seizure was diagnosed by the treating physician by a compatible anamnesis or observation of a seizure. Previous studies showed that 8–29% of patients presenting to the emergency room with clinically suspected seizures are eventually classified as having Psychogenic Non-epileptic Seizures (PNES) [40–42]. In our study only two patients received a final diagnosis of PNES. Potentially, more patients were misclassified as having a seizure. However, as an altered mental status was present in a large proportion of patients (post-ictal phase) which is more common in epileptic seizures than in PNES [43, 44], it is unlikely that this considers a substantial number of patients. Lastly, in this study approximately 5–10% of all patients eligible for inclusion did not give consent for participation. Considering this small proportion, we assume that selection bias did not influence results.

## Conclusions

In conclusion, in patients suspected of a CNS infection presenting with a seizure, approximately one in five was diagnosed with a CNS infection, and almost half showed elevated CSF leukocyte count. CSF changes in epileptic seizures of uncertain etiology were uncommon and could not be attributed to ictal activity alone. The best predictor for CNS infection in this population was CSF leukocyte count, and diagnostic accuracy of other clinical and laboratory features was low. Therefore, these characteristics cannot be used to rule out CNS infection.

## Data Availability

The data that support the findings of this study are available on request from the corresponding author. The data are not publicly available due to privacy or ethical restrictions.

## Abbreviations

AUC: Area under the curve
CNS: Central nervous system
CRF: Case record form
CSF: Cerebrospinal fluid
CT: Computed tomography
EEG: Electroencephalography
GCS: Glasgow coma scale
GOS: Glasgow outcome scale
HIV: Human immunodeficiency virus
HSV: Herpes simplex virus
IQR: Interquartile range
JCV: John Cunningham virus
MRI: Magnetic resonance imaging
NPV: Negative predictive value
PCR: Polymerase chain reaction
PML: Progressive multifocal leukoencephalopathy
PNES: Psychogenic non-epileptic seizures
PPV: Positive predictive value
ROC: Receiver operating characteristic
VZV: Varicella-zoster virus
